# The association between creatinine to body weight ratio and the risk of progression to diabetes from pre-diabetes: a 5-year cohort study in Chinese adults

**DOI:** 10.1186/s12902-023-01518-9

**Published:** 2023-12-04

**Authors:** Tong Li, Changchun Cao, Xuan Xuan, Wenjing Liu, Xiaohua Xiao, Cuimei Wei

**Affiliations:** 1 Department of Nephrology, Shenzhen Second People’s Hospital, Shenzhen, Guangdong Province 518000 China; 2 Department of Nephrology, The First Affiliated Hospital of Shenzhen University, Shenzhen, Guangdong Province 518000 China; 3 Department of Rehabilitation, Shenzhen Dapeng New District Nan’ao People’s Hospital, Shenzhen, Guangdong Province 518000 China; 4 Department of Rheumatology, Shenzhen Second People’s Hospital, Shenzhen, Guangdong Province 518000 China; 5 Department of Rheumatology, The First Affiliated Hospital of Shenzhen University, Shenzhen, Guangdong Province 518000 China; 6 Department of Geriatrics, Shenzhen Second People’s Hospital, No.3002 Sungang Road, Futian District, Shenzhen, Guangdong Province 518000 China; 7 Department of Geriatrics, The First Affiliated Hospital of Shenzhen University, Shenzhen, Guangdong Province 518000 China

**Keywords:** Pre-diabetes, Diabetes, Non-linear relationship, Creatinine to body weight ratio, Cox proportional-hazards regression

## Abstract

**Objective:**

Evidence on the association between the creatinine to body weight (Cre/BW) ratio and the risk of pre-diabetes to diabetes development remains limited. Our study aimed to examine the association between the Cre/BW ratio and incident diabetes in pre-diabetic patients.

**Methods:**

This retrospective cohort study included 24,506 pre-diabetic participants who underwent health checks from 2010 to 2016 in China. We used the Cox proportional-hazards regression model to explore the relationship between baseline Cre/BW ratio and diabetes risk in pre-diabetes patients. Using a Cox proportional hazards regression with cubic spline function and smooth curve fitting (cubical spline smoothing), we were able to determine the non-linear relationship between them. We also carried out a number of subgroup and sensitivity analyses.

**Results:**

The age range of the participants included in this study was 20–99 years, with a majority of 16,232 individuals (66.24%) being men. The mean baseline Cre/BW ratio was 1.06 (SD 0.22) umol/L/kg. 2512 (10.25%) participants received a diabetes final diagnosis over a median follow-up period of 2.89 years. After adjusting for covariates, the Cre/BW ratio had a negative association with incident diabetes in participants with pre-diabetes, per umol/L/kg increase in Cre/BM ratio was accompanied by a 55.5% decrease in diabetes risk (HR = 0.445, 95%CI 0.361 to 0.548). The Cre/BW ratio and risk of diabetes had a non-linear connection, with 1.072 umol/L/kg serving as the ratio's inflection point. The HR were 0.294 (95%CI:0.208–0.414) and 0.712 (95%CI:0.492–1.029), respectively, on the left and right sides of the inflection point. The sensitivity analysis demonstrated the robustness of these results. Subgroup analyses indicated that the Cre/BW ratio was strongly associated with the risk of diabetes among participants who were younger than 50 years, as well as among those with diastolic blood pressure (DBP) < 90 mmHg and triglyceride (TG) < 1.7 mmol/L. In contrast, among participants 50 years of age or older, those with DBP ≥ 90 mmHg, and those with TG ≥ 1.7 mmol/L, the relationship between the Cre/BW ratio and the risk of diabetes was attenuated.

**Conclusion:**

This study demonstrates a negative, non-linear relationship between the Cre/BW ratio and the risk of diabetes among the Chinese population with pre-diabetes. From a therapeutic standpoint, it is clinically meaningful to maintain the Cre/BW ratio levels above the inflection point of 1.072 umol/L/kg.

**Supplementary Information:**

The online version contains supplementary material available at 10.1186/s12902-023-01518-9.

## Introduction

Diabetes mellitus (DM) is a worldwide epidemic that has been linked to significant financial expenditures. China has the most considerable percentage of diabetic patients worldwide as of 2013. By 2035, it is expected to have the greatest impact [1]. According to the International Diabetes Federation (IDF), the global prevalence of diabetes among 20–79-year-olds was expected to be 10.5% (536.6 million individuals) in 2021 and will increase to 12.2% (783.2 million) in 2045 [2]. Diabetes increases the risk of cardiovascular disease and is associated with relatively specific microvascular problems affecting the eyes, nerves, and kidneys [3–5]. DM with diabetic complications is becoming a crucial public health issue. Therefore, only by fully understanding the risk factors of diabetes can we effectively and timely prevent and screen diabetes.

Pre-diabetes, which includes impaired fasting glucose (IFG) and impaired glucose tolerance, is the transition from normal glucose metabolism to diabetes (IGT) [6]. A recent national cross-sectional survey revealed that 35.7% of Chinese adults had pre-diabetes [7]. In those with pre-diabetes, the annual chance of getting diabetes is 5–10%, and up to 70% of people eventually develop the disease [8]. Interventions initiated at the pre-diabetes stage are more effective and prudent than those initiated after the beginning of diabetes because they can stop or delay pre-diabetes progression to diabetes [9, 10]. Considering that patients with pre-diabetes are more likely to develop diabetes than the general population, it is even more urgent to explore the risk factors for progression from pre-diabetes to diabetes and to intervene promptly.

Skeletal muscle is one of insulin's primary target organs and plays an important role in maintaining glucose homeostasis [11, 12]. According to a recent study, decreasing muscle mass was closely associated with insulin resistance (IR) [13]. Skeletal muscle atrophy and abnormal muscle protein metabolisms have been seen in people with type 2 diabetes mellitus (T2DM). Creatinine (Cre) is the only phosphate creatine metabolite found in the body's skeletal muscles. Cre, despite being a measure of renal function, is affected by muscle size because it is produced by muscle mass. Due to the stability of total skeletal muscle mass, the Cre concentration is also reasonably steady [14]. Serum creatinine (Scr) is regarded as a low-cost and simple-to-measure index for assessing the condition of skeletal muscle [15]. Recent research has revealed that the intriguing new biomarker of Cre to body weight ratio (Cre/BW) is strongly associated with incident DM [16, 17] and non-alcoholic fatty liver disease (NAFLD) [18, 19] in general populations. Furthermore, recent research found that the Cre/BW ratio is closely related to the incidence of diabetes in general Chinese participants [20]. However, the relationship between the Cre/BW ratio and DM has not been studied in pre-diabetes participants, a population that is prone to progress to diabetes. Therefore, in order to ascertain the connection between the Cre/BW ratio and the risk of developing diabetes from pre-diabetes, we carried out a retrospective cohort analysis utilizing available data.

## Methods

### Study design

Using information from the database provided by China Rich Healthcare Group, we carried out a retrospective cohort research. The Cre/BW ratio at baseline was the most interesting independent variable in the present study. The dependent variable was diabetes diagnosed during follow-up (dichotomous variable: 0 = non-DM, 1 = DM).

### Data source

The data used in the study is from a publicly available database. The raw data was taken from the DATADRYAD database (https://datadryad.org/stash) for free provided by Chen, Ying et al. (2018), Data from: Association of body mass index and age with incident diabetes in Chinese adults: a population-based cohort study, Dryad, Dataset, https://doi.org/10.5061/dryad.ft8750v. Researchers could use this data for secondary studies without breaching the rights of the authors in accordance with Dryad's terms of service [21].

### Study population

To mitigate the impact of selection bias, a non-selective and consecutive collection of participant data was conducted at 32 sites across 11 cities in China (including Beijing, Suzhou, Changzhou, Shanghai, Nantong, Shenzhen, Chengdu, Nanjing, Guangzhou, Hefei, and Wuhan). This approach aimed to minimize the potential for over- or under-estimation of results, which is a common issue associated with selection bias in studies. To protect the anonymity of the participants, their identification information was encoded using non-traceable codes. The China Rich Healthcare Group's electronic medical record system was used to retrieve the data. The Rich Healthcare Group Review Board approved the original study, and the informed consent was waived because of the retrospective nature of this study [22].

**Fig. 1:**
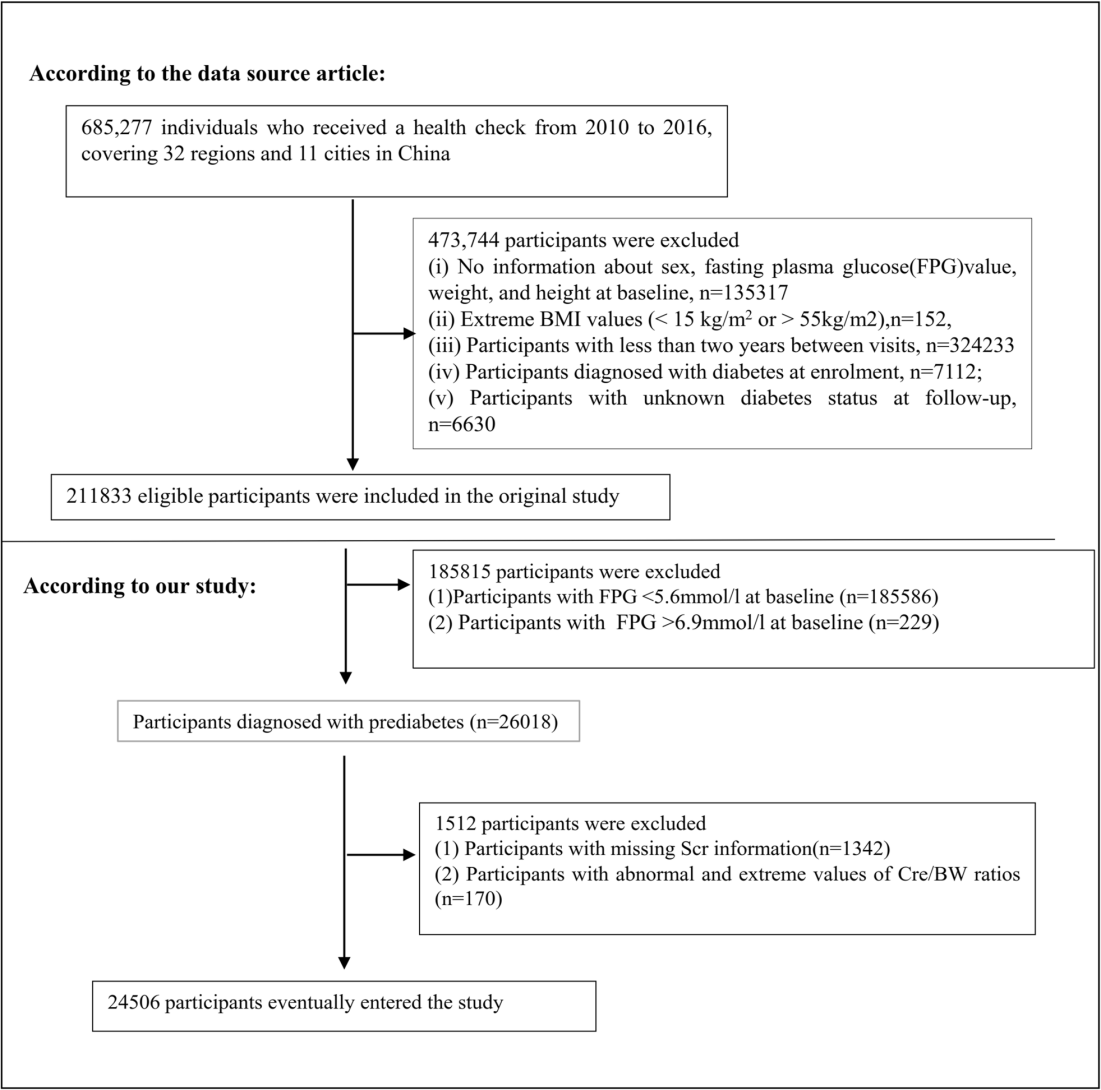
Flowchart of study participants. Figure 1 showed the inclusion of participants. In the original study, 211,833 participants were assessed for eligibility. We further excluded 187,327 participants. The final analysis included 24,506 subjects in the present study

The original study comprised 685,277 people who were at least 20 years old and had at least two health checks. The original study removed 473,744 subjects in total. Ultimately, 211,833 people were included in the initial research analysis. The following were the exclusion criteria for the original study: (i) absence of fasting plasma glucose (FPG) value, weight, sex, and height at baseline; (ii) extreme body mass index (BMI) values (< 15 kg/m^2^ or > 55 kg/m^2^); (iii) fewer than two years of follow-up times; (iv) diagnosis of diabetes at enrollment; and (v) uncertain diabetes status at follow-up (18). We first excluded 185,815 participants who were outside the FPG 5.6–6.9 mmol/l range at baseline in the current study. Pre-diabetes was characterized as an FPG level between 5.6 and 6.9 mmol/L by the American Diabetes Association 2021 guidelines [23]. We further excluded participants with missing Scr information (*n* = 1352) and those with abnormal and extreme Cre/BW ratio (greater or less than three standard deviations from the mean) (*n* = 170) [24]. The secondary analysis eventually comprised 24,506 subjects. Figure 1 depicted the process of participant selection.

### Variables

#### Cre to body weight ratio

We obtained the information on the Cre to body weight ratio at baseline and recorded it as a continuous variable. Defining the Cre/BW ratio was described as: Cre/BW ratio = serum creatinine divided by body weight. Notably, the unit of Cre was umol/L, and the unit of BW was kg.

#### Outcome measures

Our primary outcome variable was incident diabetes (dichotomous variable: 0 = non-DM, 1 = DM). At the follow-up evaluation, incident diabetes was diagnosed based on self-report or FPG ≥ 7.0 mmol/l [21]. Participants were censored at the time of diabetes diagnosis or their last visit, whichever came first. The duration of follow-up was five years.

#### Covariates

We chose covariates for our study based on previous research and clinical experience [16, 17, 20, 21]. The following variables were therefore used as covariates based on the principles outlined above (1) continuous variables: systolic blood pressure (SBP), height, age, Blood urea nitrogen(BUN), diastolic blood pressure (DBP), triglyceride(TG), FPG, total cholesterol (TC), alanine aminotransferase(ALT), high-density lipoprotein cholesterol (HDL-c), aspartate aminotransferase(AST), low-density lipoprotein cholesterol(LDL-c); (2) categorical variables: smoking status, gender, drinking status, and family history of diabetes.

#### Variable measurement

Each time they visited the health check center, each participant was given a thorough questionnaire that inquired about their lifestyle, family history of chronic illness, demographic factors, and medical history. Weight, blood pressure, and height were measured by trained staff. Participants were weighed in light clothing with no shoes and measured to nearest 0.1 kg. Height was measured accurately to within 0.1 cm. Blood pressure was measured using mercury sphygmomanometers. Fasting venous blood samples were collected after fasting for at least 10 h at each appointment. HDL-c, AST, Scr, FPG, TC, BUN, TG, ALT, and LDL-c were measured on an autoanalyzer (Beckman 5800) [21].

#### Missing data processing

In observational research, missing data were common and could not be prevented entirely [25]. In our study, the number of participants with missing data of SBP, DBP, TC, TG, HDL-c, LDL-c, ALT, AST, BUN, smoking status, and drinking status was 6(0.024%), 6(0.024%), 394(1.61%), 394(1.61%), 9299(37.95%), 8683(35.43%), 133(0.54%), 13,822(56.40%), 1559(6.36%), 16,184(66.04%), and 16,184(66.04%), respectively. To reduce the variation brought on by missing variables, which made it impossible to accurately depict the statistical effectiveness of the target sample throughout the modeling phase, this study used multiple imputations [26]. The imputation model included age, sex, height, TG, AST, DBP, BUN, TC, ALT, LDL-c, smoking status, HDL-c, drinking status, SBP, and family history of diabetes. The procedures for analyzing missing data used missing-at-random (MAR) assumptions [25].

### Statistical analysis

Quartiles and deciles of the Cre/BW ratio stratified the participants. In the case of continuous variables, baseline characteristics were presented as mean ± standard deviation (SD) (Gaussian distribution) or median (range) (Skewed distribution), and as percentages for categorical variables. We used three kinds of statistical tests to detect the differences among different Cre/BW ratio groups: χ2 for categorical variables, One-Way ANOVA for normal distribution, or Kruskal-Whallis H for skewed distribution. We employed the Kaplan–Meier method to compute the survival estimates and time-to-event variables. We utilized the log-rank test in pre-diabetes populations to compare the probability of diabetes-free survival among Cre/BW ratio groups [27].

To assess covariate colinearity, the variance inflation factor (VIF) was calculated [28]. VIF = 1/(1-R^2^). Where R^2^ was the R-squared value derived from a linear regression equation, this variable was the dependent variable, and all other variables were independent. If the VIF was greater than 5, then the variables would be considered collinear and could not be included in the multiple regression model (Table S1).

Following collinearity screening, we used the univariate and multivariate Cox proportional-hazards regression to assess three alternative models for the relationship between the Cre/BW ratio and the risk of diabetes. As for the model I, it was the nonadjusted model with no covariates adjusted. As for model II, it was the minimally-adjusted model with only sociodemographic variables adjusted, including SBP, gender, DBP, age, family history of diabetes, height, drinking, and smoking status. Model III was the fully-adjusted model with covariates presented in Table 1, including SBP, gender, FPG, age, height, BUN, DBP, TG, ALT, HDL-c, AST, family history of diabetes, LDL-c, drinking, and smoking status. In cases where the Hazard ratio (HR) increased by 10% or more after covariances were introduced to the model, we recorded the effect sizes (HR) and 95% confidence intervals (CIs) and adjusted them [29]. Additionally, it referred to the outcomes of the collinearity screening. We ultimately decided not to include TC in the multivariate Cox proportional-hazards regression equation since the results of the collinearity screening indicated that it was collinear with other variables (Table S1).

**Table 1:** The baseline characteristics of participants

Cre/BW ratio group	< 0.9	0.9–1.04	1.04–1.20	≥ 1.20
**Participants**	6127	6126	6126	6127
**Age(years)**	47.8 ± 12.4	49.1 ± 13.0	49.4 ± 13.9	50.9 ± 15.5
**Height(cm)**	166.9 ± 8.9	167.1 ± 8.5	167.1 ± 8.1	165.9 ± 7.8
**Weight(kg)**	75.8 ± 13.4	70.7 ± 11.5	67.9 ± 10.4	62.9 ± 9.4
**BMI(kg/m**^**2**^**)**	27.1 ± 3.5	25.2 ± 2.9	24.2 ± 2.7	22.8 ± 2.7
**SBP(mmHg)**	128.9 ± 17.6	127.1 ± 17.6	126.2 ± 17.0	126.5 ± 18.0
**DBP(mmHg)**	79.6 ± 11.4	78.7 ± 11.1	78.0 ± 10.9	77.2 ± 11.0
**FPG(mmol/L)**	6.0 ± 0.3	6.0 ± 0.3	5.9 ± 0.3	5.9 ± 0.3
**TC(mmol/L)**	5.0 ± 1.0	5.0 ± 1.0	5.0 ± 1.0	4.9 ± 0.9
**TG(mmol/L)**	1.6 (1.1–2.4)	1.5 (1.0–2.2)	1.4 (1.0–2.1)	1.3 (0.9–1.9)
**HDL-c(mmol/L)**	1.3 ± 0.3	1.3 ± 0.3	1.3 ± 0.3	1.4 ± 0.3
**LDL-c(mmol/L)**	2.9 ± 0.7	2.9 ± 0.7	2.9 ± 0.7	2.9 ± 0.7
**ALT(U/L)**	25.0 (17.0–39.4)	23.0 (15.8–35.0)	22.0 (15.3–32.4)	19.5 (14.3–27.7)
**AST(U/L)**	28.0 ± 13.4	26.7 ± 13.0	26.0 ± 11.1	24.9 ± 9.9
**BUN(mmol/L)**	4.7 ± 1.2	4.9 ± 1.2	5.0 ± 1.2	5.2 ± 1.3
**Scr (umol/L)**	59.8 ± 11.1	68.9 ± 11.3	75.9 ± 11.7	85.3 ± 13.2
**Cre/BW ratio(umol/L/kg)**	0.8 ± 0.1	1.0 ± 0.0	1.1 ± 0.0	1.4 ± 0.1
**Gender**				
Male	3058 (49.9%)	3896 (63.6%)	4468 (72.9%)	4810 (78.5%)
Female	3069 (50.1%)	2230 (36.4%)	1658 (27.1%)	1317 (21.5%)
**Smoking status**				
Never smoker	4782 (78.0%)	4470 (73.0%)	4299 (70.2%)	4233 (69.1%)
Ever smoker	228 (3.7%)	261 (4.3%)	294 (4.8%)	327 (5.3%)
Current smoker	1117 (18.2%)	1395 (22.8%)	1533 (25.0%	1567 (25.6%)
**Drinking status**				
Never drinker	5143 (83.9%)	4931 (80.5%)	4793 (78.2%)	4837 (78.9%)
Ever drinker	792 (12.9%)	957 (15.6%)	1077 (17.6%)	1020 (16.6%)
Current drinker	192 (3.1%)	238 (3.9%)	256 (4.2%)	270 (4.4%)
**Family history of diabetes**				
No	5909 (96.4%)	5950 (97.1%)	6008 (98.1%)	6020 (98.3%)
Yes	218 (3.6%)	176 (2.9%)	118 (1.9%)	107 (1.7%)

Values are n(%), mean ± SD or medians (quartiles)

*BMI* Body mass index, *FPG* Fasting plasma glucose, *DBP* Diastolic blood pressure, *TC* Total cholesterol, *SBP* Systolic blood pressure, *TG* Triglyceride, *ALT* Alanine aminotransferase, *LDL-c* Low-density lipid cholesterol, *AST* Aspartate aminotransferase, *HDL-c* High-density lipoprotein cholesterol, *BUN* Blood urea nitrogen, *Scr* Serum creatinine, *Cre/BW ratio* creatinine to body weight ratio

Methods based on Cox proportional-hazards regression models were often accused of being unsuitable for dealing with non-linear models. As a result, we applied the Cox proportional hazards regression model with cubic spline functions and the smooth curve fitting (penalized spline method) to address the non-linearity between the Cre/BW ratio and diabetes. Following the detection of non-linearity, the inflection point was computed using a recursive technique, and two-piecewise Cox proportional-hazards regression models were conducted on both sides of the inflection point. The optimal model for characterizing the risk associated with the Cre/BW ratio and diabetes was determined using a log-likelihood ratio test [30].

For subgroup analysis, a stratified Cox proportional-hazards regression model was applied to the various subgroups (gender, BMI, age, SBP, TG, DBP, drinking and smoking status, and family history of diabetes). Firstly, continuous variable age (< 30, ≥ 30 to < 40, ≥ 40 to < 50, ≥ 50 to < 60, ≥ 60 to < 70, ≥ 70 years), BMI (< 18.5, ≥ 18.5 to < 24, ≥ 24 to 28, ≥ 28 kg/m^2^), SBP(< 140, ≥ 140 mmHg), DBP(< 90, ≥ 90 mmHg), TG(< 1.7, ≥ 1.7 mmol/L) were converted to a categorical variable based on the clinical cut point [31–33]. Secondly, we adjusted each stratification for all factors in addition to the stratification factor itself (SBP, gender, FPG, age, height, BUN, DBP, TG, ALT, HDL-c, AST, family history of diabetes, LDL-c, drinking and smoking status). Lastly, a likelihood ratio test for models with and without interaction terms was used to test for interactions [34, 35].

We conducted a sensitivity analysis of our findings to determine their reliability. To confirm the results of the Cre/BW ratio as a continuous variable and investigate the possibility of non-linearity, the Cre/BW ratio was converted into a categorical variable in accordance with the deciles. Obesity and weight gain are associated with an increased risk of developing diabetes [36]. Smoking and alcohol consumption are related to an increased risk of T2DM [37]. When exploring the Cre/BW ratio and incident diabetes association in other sensitivity analyses, we excluded participants with a smoking and drinking history. We also excluded drinking and smoking status from the multivariate model as sensitivity analysis. Smoking and drinking status did not have complete data in about 70% of the cases, and might not be useful as covariates to adjust in the model. Furthermore, the continuity covariate was also inserted into the equation (model IV) as a curve using cubic splines to ensure the robustness of the results [38]. We also calculated E-values to examine the possibility of unmeasured confounding between the Cre/BW ratio and the risk of diabetes [39]. The STROBE statement was used to write all the results [29].

R (http://www.r-project.org, The R Foundation) and EmpowerStats (http://www.empowerstats.com, X&Y Solutions, Inc., Boston, MA) were used to execute the statistical analyses. A *P*-value of < 0.05 determined statistical significance in all cases.

## Results

### Characteristics of participants

Table 1 listed the baseline characteristics of the participating participants. The age range of the participants included in this study was 20–99 years, with a majority of 16,232 individuals (66.24%) being men. The mean baseline Cre/BW ratio was 1.06 (SD 0.22) umol/L/kg. During a median follow-up period of 2.89 years, 2512 patients (10.25%) were diagnosed with diabetes. We assigned adults into subgroups using Cre/BW ratio quartiles (Q1: < 0.90 umol/L/kg, Q2: 0.90–1.04 umol/L/kg, Q3: 1.04–1.20 umol/L/kg, Q4: ≥ 1.20 umol/L/kg). When compared with the Q1(< 0.90 umol/L/kg) group, the values or proportions of age, HDL-c, BUN, Scr, males, current or ever smokers, and current or ever drinkers increased significantly in the Q4(Cre/BW ratio ≥ 1.20 umol/L/kg) group. In contrast, the opposite results were detected in covariates in terms of SBP, LDL-c, BMI, height, weight, FPG, TG, AST, DBP, TC, ALT, females, never smokers, never drinkers, and family history of diabetes.

**Fig. 2:**
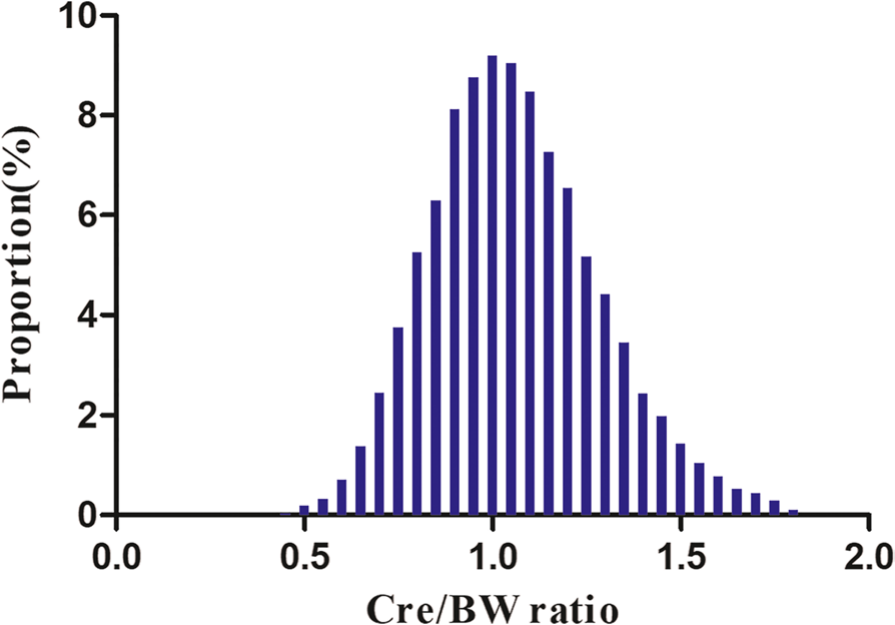
Distribution of the Cre/BW ratio. Figure 2. It presented a normal distribution ranging from 0.377 to 1.802 umol/L/kg, and the average was 1.062 umol/L/kg

**Fig. 3:**
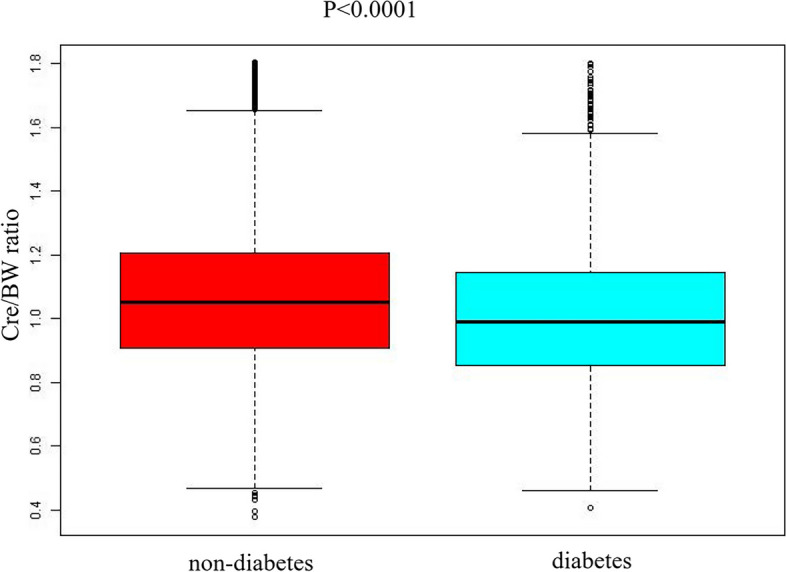
Data visualization of the Cre/BW ratio of all participants from diabetes and non-diabetes groups. Figure 3 revealed that the Cre/BW ratio distribution level was lower in the diabetic group. In contrast, the ratio of Cre to BW was considerably larger in the non-diabetic group

**Fig. 4:**
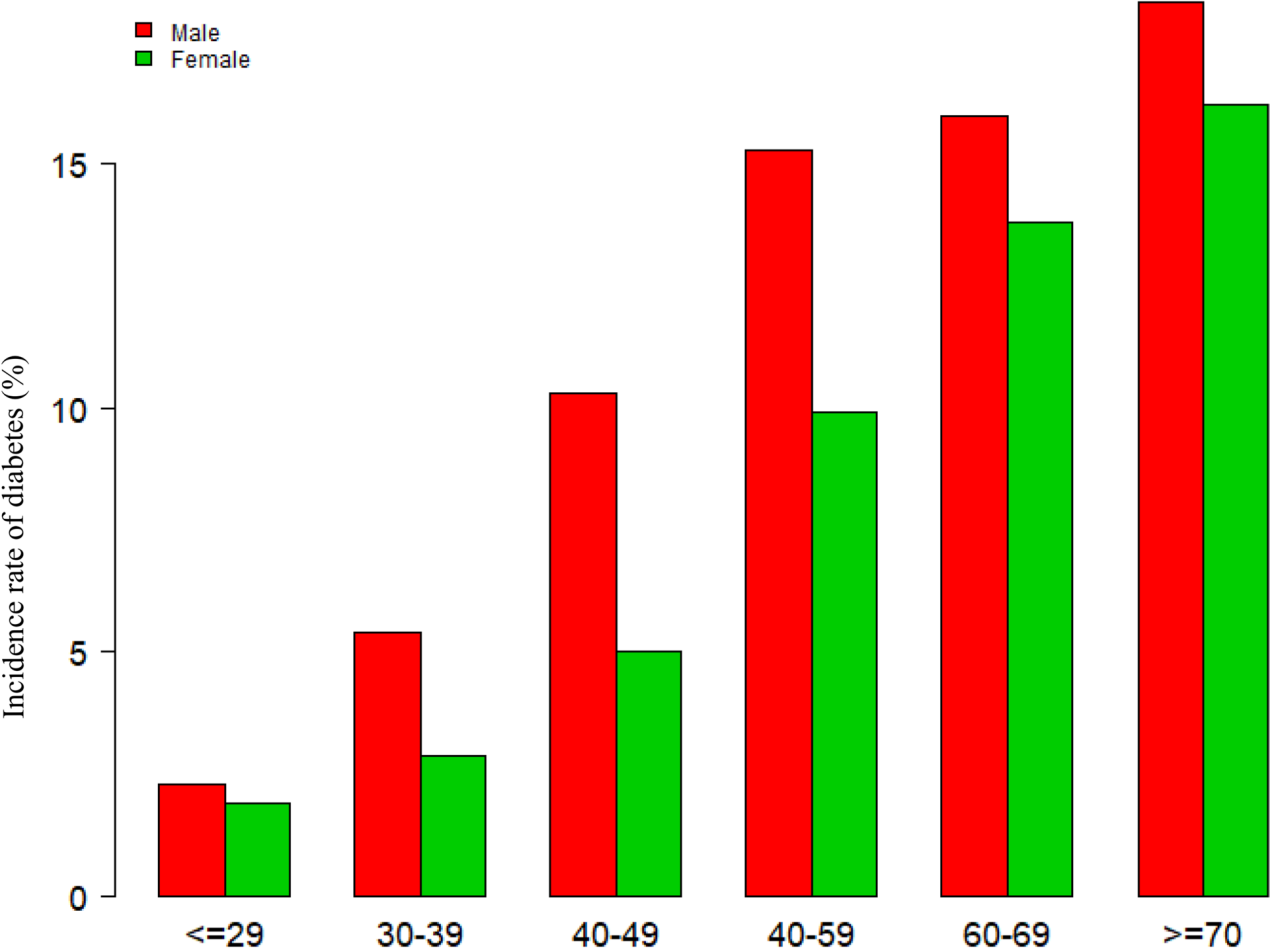
Diabetes incidence rate of age stratification by 10 intervals. Figure 4 demonstrated that, regardless of age group, male individuals had a higher incidence of diabetes than female subjects in age stratification by ten intervals. It was also shown that the prevalence of diabetes increased with age in both men and women

According to Fig. 2, the Cre/BW ratio levels had a normal distribution ranging from 0.377 to 1.802 umol/L/kg; the average was 1.062 umol/L/kg. Participants were divided into two groups based on whether participants progressed to diabetes from pre-diabetes during the follow-up. As shown in Fig. 3, the distributions of the Cre/BW ratio in the non-diabetes group were higher than those in the diabetes group. Men were more likely to progress to diabetes regardless of age group when age groups were stratified by 10 intervals (Fig. 4). Furthermore, the incidence of diabetes increased with age in both females and males.

### The incidence rate of diabetes in participants with pre-diabetes

**Fig. 5:**
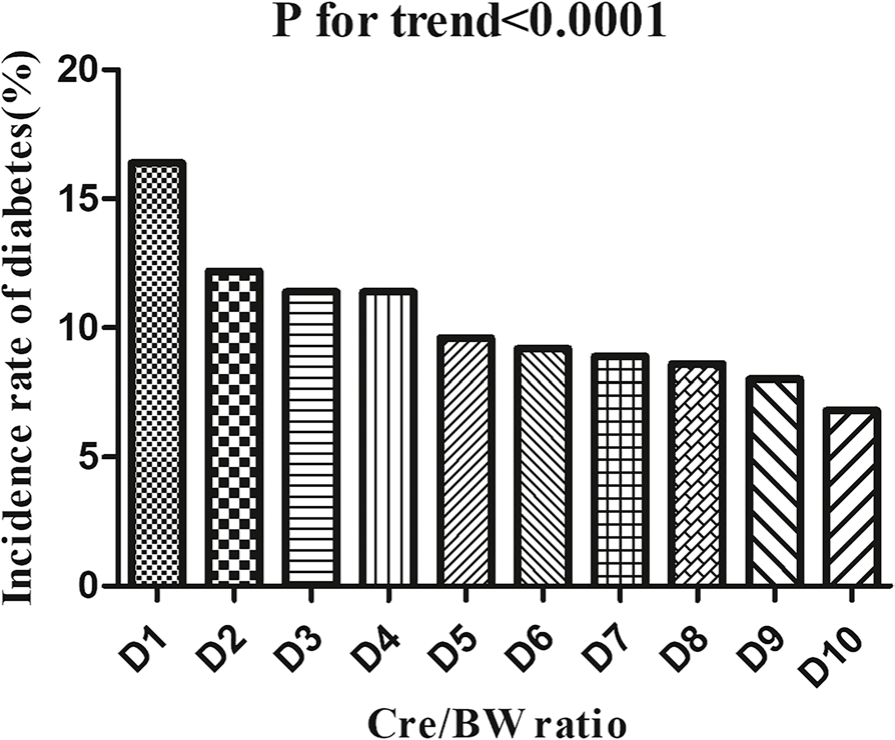
Incidence of diabetes according to the deciles of the Cre/BW ratio. Figure 5. Participants in the high Cre/BW ratio group had a lower diabetes incidence than the lower Cre/BW ratio group (*P* < 0.0001 for trend)

Table 2 revealed that 2512 (10.25%) participants progressed to diabetes from pre-diabetes during a median follow-up time of 2.89 years. Incidence rates for all people combined were 3.47 per 100 person-years. Particularly, the four Cre/BW ratio groups had cumulative incidences of 4.62, 3.62, 3.07, and 2.57 per 100 person-years, respectively. The incidence rate of total diabetes and each Cre/BW ratio group was 10.25%(9.87%-10.63%), 13.68%(12.82%-14.54%), 10.71%(9.93%-11.48%), 9.09%(8.37%-9.81%), and 7.52%(6.86%-8.18%), respectively. Participants with a high Cre/BW ratio had lower incidence rates of diabetes compared to the group with the lowest Cre/BW ratio (*P* < 0.0001 for trend) (Fig. 5).

**Table 2:** Incidence rate of incident diabetes

Cre/BW ratio	Participants (n)	diabetes events (n)	Incidence rate (95% CI) (%)	Cumulative incidence (Per 100 person-year)
Total	24,506	2512	10.25 (9.87–10.63)	3.47
D1	2451	401	16.36 (14.90–17.83)	5.53
D2	2450	298	12.16 (10.87–13.46)	4.10
D3	2450	280	11.43 (10.17–12.69)	3.85
D4	2451	279	11.38 (10.12–12.64)	3.85
D5	2451	236	9.63 (8.46–10.80)	3.26
D6	2458	226	9.19 (8.05–10.34)	3.06
D7	2442	218	8.93 (7.80–10.06)	3.06
D8	2451	212	8.65 (7.54–9.76)	2.93
D9	2451	195	7.96 (6.88–9.03)	2.72
D10	2451	167	6.81 (5.81–7.81)	2.34

*Cre/BW ratio* Creatinine to body weight ratio

### The results of univariate analyses using the Cox proportional-hazards regression model

The univariate analysis was conducted on the available data, showing that the factors in terms of height and Scr were not connected with diabetes. Still, age, weight, BMI, SBP, DBP, FPG, TC, TG, LDL-c, HDL-c, ALT, AST, BUN, current smokers, current drinkers were positively associated to diabetes, and Cre/BW ratio, females, ever drinkers were negatively related with diabetes (See Table 3 for detail).

**Table 3:** Univariate proportional hazard analysis (*n* = 24,506)

Variable	HR (95%CI)	*P* value
**Age(years)**	1.030 (1.027, 1.033)	< 0.00001
**Gender**		
Male	Ref	
Female	0.812 (0.743, 0.886)	< 0.00001
**Height(cm)**	0.998 (0.993, 1.003)	0.40559
**Weight(kg)**	1.023 (1.020, 1.026)	< 0.00001
**BMI(kg/m**^**2**^**)**	1.108 (1.097, 1.120)	< 0.00001
**SBP(mmHg)**	1.015 (1.013, 1.017)	< 0.00001
**DBP(mmHg)**	1.017 (1.013, 1.020)	< 0.00001
**FPG(mmol/L)**	9.534 (8.643, 10.516)	< 0.00001
**TC(mmol/L)**	1.070 (1.028, 1.114)	0.00089
**TG(mmol/L)**	1.114 (1.093, 1.135)	< 0.00001
**LDL-c(mmol/L)**	1.065 (1.009, 1.123)	0.02154
**HDL-c(mmol/L)**	1.180 (1.037, 1.342)	0.01202
**ALT(U/L)**	1.005 (1.004, 1.006)	< 0.00001
**AST(U/L)**	1.011 (1.009, 1.012)	< 0.00001
**BUN(mmol/L)**	1.037 (1.004, 1.070)	0.02580
**Scr(umol/L)**	1.001 (0.998, 1.004)	0.48214
**Cre/BW ratio (umol/L/kg)**	0.387 (0.321, 0.466)	< 0.00001
**Smoking status**		
Never smoker	Ref	
Ever smoker	1.140 (0.950, 1.367)	0.15897
Current smoker	1.287 (1.179, 1.405)	< 0.00001
**Drinking status**		
Never drinker	Ref	
Ever drinker	0.895 (0.801, 0.999)	0.04731
Current drinker	1.279 (1.075, 1.522)	0.00547
**Family history of diabetes**		
No	Ref	
Yes	1.435 (1.181, 1.743)	0.00027

*BMI* Body mass index, *FPG* Fasting plasma glucose, *DBP* Diastolic blood pressure, *TC* Total cholesterol, *SBP* Systolic blood pressure, *TG* Triglyceride, *ALT* Alanine aminotransferase, *LDL-c* Low-density lipid cholesterol, *AST* Aspartate aminotransferase, *HDL-c* High-density lipoprotein cholesterol, *BUN* Blood urea nitrogen, *Scr* Serum creatinine, *Cre/BW ratio* Creatinine to body weight ratio, *HR* Hazard ratios, *CI* Confidence interval, *Ref* Reference

**Fig. 6:**
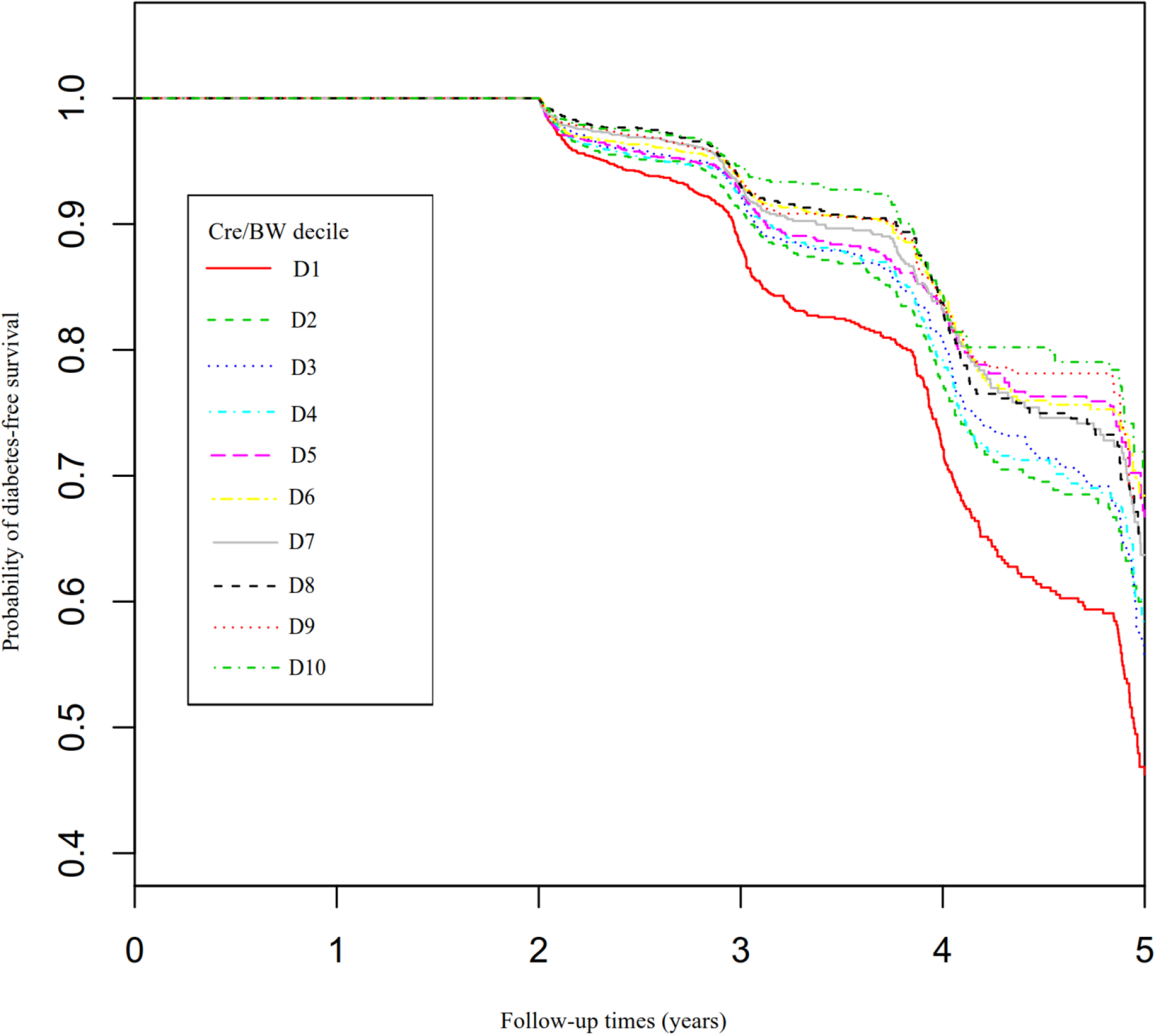
Kaplan–Meier event-free survival curve. Figure 6. Kaplan–Meier event-free survival curve. The probability of diabetes-free survival differed significantly between the Cre/BW ratio groups (log-rank test, *P* < 0.0001). The probability of diabetes-free survival gradually increased with the rising Cre/BW ratio, indicating that the group with the highest Cre/BW ratio had the lowest risk of developing diabetes

Figure 6 depicted the Kaplan–Meier survival curves for diabetes-free survival probability stratified by Cre/BW ratio group. Between the Cre/BW ratio groups, there were substantial differences in the likelihood of surviving without developing diabetes (log-rank test, *P* < 0.0001). Diabetes-free survival probabilities increased as the Cre/BW ratio increased, which indicated that those with the highest Cre/BW ratio faced the lowest risk of diabetes.

### Results from a multivariate Cox proportional-hazards regression model

The authors created three models using the Cox proportional-hazards regression model to examine the relationship between the Cre/BW ratio and diabetes in the subjects with pre-diabetes. In the unadjusted model (Model I), a 61.3% reduction in the risk of diabetes was associated with an increase of 1 unit (umol/L/kg) in the Cre/BW ratio (HR = 0.387, 95%CI 0.321 to 0.466). Statistical significance was found in the results. When only demographic factors were considered in the minimally-adjusted model (Model II), the risk of diabetes fell by 76.5% for each extra umol/L/kg of the Cre/BW ratio (HR = 0.235, 95%CI 0.191 to 0.288). The model's results on the relationship between diabetes and the Cre/BW ratio were statistically significant. In the fully-adjusted model (Model III), each additional umol/L/kg of the Cre/BW ratio was accompanied by a 55.5% decrease in diabetes risk (HR = 0.445, 95%CI 0.361 to 0.548). As shown by the distribution of confidence intervals, the relationship between the Cre/BW ratio and diabetes obtained by the model was reliable (Table 4).

**Table 4:** Relationship between Cre/BW ratio and the incident diabetes in different models

Exposure	Model I (HR,95%CI,P)	Model II (HR,95%CI,P)	Model III (HR,95%CI,P)	Model IV (HR,95%CI, P)
**Cre/BW ratio**	0.387 (0.321, 0.466) < 0.0001	0.235 (0.191, 0.288) < 0.0001	0.445 (0.361, 0.548) < 0.0001	0.521 (0.420, 0.646) < 0.0001
**Cre/BW ratio Deciles**				
**D1**	Ref	Ref	Ref	Ref
**D2**	0.744 (0.640, 0.864) 0.00011	0.695 (0.597, 0.808) < 0.0001	0.817 (0.701, 0.951) 0.00902	0.819 (0.703, 0.955) 0.01063
**D3**	0.704 (0.604, 0.820) < 0.0001	0.624 (0.535, 0.728) < 0.0001	0.742 (0.635, 0.867) 0.00017	0.750 (0.641, 0.877) 0.00032
**D4**	0.708 (0.608, 0.825) < 0.0001	0.607 (0.519, 0.709) < 0.0001	0.768 (0.656, 0.899) 0.00102	0.772 (0.659, 0.905) 0.00143
**D5**	0.595 (0.507, 0.699) < 0.0001	0.508 (0.432, 0.599) < 0.0001	0.671 (0.568, 0.792) < 0.0001	0.692 (0.585, 0.818) 0.00002
**D6**	0.539 (0.458, 0.635) < 0.0001	0.450 (0.380, 0.532) < 0.0001	0.574 (0.485, 0.680) < 0.0001	0.608 (0.512, 0.722) < 0.0001
**D7**	0.580 (0.492, 0.685) < 0.0001	0.464 (0.392, 0.551) < 0.0001	0.666 (0.560, 0.792) < 0.0001	0.703 (0.591, 0.837) 0.00007
**D8**	0.544 (0.461, 0.643) < 0.0001	0.422 (0.355, 0.503) < 0.0001	0.614 (0.515, 0.733) < 0.0001	0.644 (0.538, 0.770) < 0.0001
**D9**	0.533 (0.449, 0.633) < 0.0001	0.386 (0.323, 0.462) < 0.0001	0.582 (0.484, 0.700) < 0.0001	0.643 (0.534, 0.775) < 0.0001
**D10**	0.472 (0.394, 0.565) < 0.0001	0.319 (0.264, 0.387) < 0.0001	0.523 (0.429, 0.637) < 0.0001	0.584 (0.477, 0.714) < 0.0001
**Age**		1.031 (1.027, 1.034) < 0.0001	1.024 (1.020, 1.027) < 0.0001	
**Gender**				
Male		Ref	Ref	
Female		0.603 (0.527, 0.691) < 0.0001	0.790 (0.687, 0.908) 0.00088	
**Height(cm)**		0.989 (0.982, 0.996) 0.00334	0.996 (0.988, 1.003) 0.21579	
**SBP(mmHg)**		1.005 (1.002, 1.008) 0.00054	1.001 (0.999, 1.004) 0.33945	
**DBP(mmHg)**		1.003 (0.999, 1.008) 0.15860	1.003 (0.998, 1.007) 0.22657	
**Smoking status**				
Never smoker		Ref	Ref	
Ever smoker		1.191 (0.987, 1.438) 0.06832	1.038 (0.860, 1.253) 0.69817	
Current smoker		1.134 (1.028, 1.251) 0.01200	1.117 (1.010, 1.234) 0.03054	
**Drinking status**				
Never drinker		Ref	Ref	
Ever drinker		0.899 (0.801, 1.010) 0.07255	0.954 (0.850, 1.071) 0.42675	
Current drinker		0.958 (0.801, 1.147) 0.64185	0.834 (0.694, 1.003) 0.05340	
**Family history of diabetes**				
No		Ref	Ref	
Yes		1.687 (1.386, 2.053) < 0.0001	1.566 (1.286, 1.907) < 0.0001	
**FPG(mmol/L)**			7.333 (6.614, 8.131) < 0.0001	
**TG(mmol/L)**			1.052 (1.028, 1.077) < 0.0001	
**LDL-c(mmol/L)**			0.941 (0.890, 0.995) 0.03138	
**HDL-c(mmol/L)**			1.591 (1.394, 1.816) < 0.0001	
**ALT(U/L)**			1.008 (1.005, 1.010) < 0.0001	
**AST(U/L)**			0.994 (0.988, 1.000) 0.04987	
**BUN(mmol/L)**			0.967 (0.935, 1.000) 0.05346	

Model I: we did not adjust other covariates

Model II: we adjust age, gender, height, SBP, DBP, family history of diabetes, smoking and drinking status

Model III: we adjust age, gender, height, SBP, DBP, FPG, BUN, TG, HDL-c, LDL-c, ALT, AST, family history of diabetes, smoking and drinking status

Model IV: we adjusted age(smooth), gender, height(smooth), SBP(smooth), DBP(smooth), FPG(smooth), BUN(smooth), TG(smooth), HDL-c(smooth), LDL-c(smooth), ALT(smooth), AST(smooth), family history of diabetes, smoking and drinking status

*HR* Hazard ratios, *CI* Confidence, *Ref* Reference, *Cre/BW ratio* Creatinine to body weight ratio

Besides, we analysed the relationship between log (Cre), log (weight) and the risk of diabetes separately (Table S2). We found that weight was positively associated with the risk of progression to diabetes in prediabetes, whereas Cre had a negative association with the risk of progression to diabetes. The results suggest that both Cre and weight contributed to our analysis of the relationship between Cre/BW ratio and diabetes.

### Sensitivity analysis

The robustness of our findings was examined using a number of sensitivity analyses. The Cre/BW ratio was first converted into a categorical variable (based on deciles), which was then included in our model after being categorical-transformed. After the Cre/BW ratio was converted into a categorical variable, the results showed that the trend of HR change was not completely consistent between the groups. The above results suggest that the relationship between the Cre/BW ratio and the risk of diabetes progression is not completely linear and that there is a possibility of a non-linear relationship between the two (Table 4).

In addition, to include the continuity covariate as a curve in the equation, we used cubic splines. This usually remained consistent with the fully adjusted model, according to the Model IV result in Table 4 (HR = 0.521, 95%CI: 0.420–0.646, *P* < 0.0001). Besides, E-values were calculated to assess sensitivity to unmeasured confounding. 3.92 was the E-value. The E-value was higher than the relative risk of unmeasured confounders and the Cre/BW ratio, implying that unmeasured or unknown confounders had little influence on the connection between the Cre/BW ratio and incident diabetes.

Furthermore, the authors excluded participants with BMI ≥ 28 kg/m^2^ (*N* = 20,431) for the sensitivity analysis. A negative association of the Cre/BW ratio with diabetes risk was also observed after adjusting for confounding factors (HR = 0.515, 95%CI:0.399 to 0.665) (Table 5). We also excluded participants with a history of smoking. The results showed that after adjusting SBP, gender, FPG, age, height, BUN, DBP, TG, ALT, HDL-c, AST, family history of diabetes, LDL-c, and drinking status, the Cre/BW ratio was still negatively associated with diabetes (HR = 0.475, 95% CI:0.370 to 0.612) (Table 5). For sensitivity analyses, we also excluded persons with a history of drinking. We still got similar results (HR = 0.453, 95% CI:0.359 to 0.571).

**Table 5:** Relationship between Cre/BW ratio and diabetes in different sensitivity analyses

Exposure	Model I (HR,95%CI, P)	Model II (HR,95%CI, P)	Model III (HR,95%CI, P)	Model IV (HR,95%CI, P)
Cre/BW ratio	0.515 (0.399, 0.665) < 0.0001	0.475 (0.370, 0.612) < 0.0001	0.453 (0.359, 0.571) < 0.0001	0.446 (0.362, 0.549) < 0.0001
Cre/BW ratio (Deciles)				
D1	Ref	Ref	Ref	Ref
D2	0.846 (0.679, 1.054) 0.13586	0.825 (0.688, 0.990) 0.03834	0.781 (0.659, 0.925) 0.00425	0.818 (0.703, 0.952) 0.00966
D3	0.753 (0.604, 0.937) 0.01116	0.787 (0.651, 0.952) 0.01346	0.767 (0.644, 0.913) 0.00289	0.743 (0.635, 0.868) 0.00018
D4	0.866 (0.699, 1.073) 0.18828	0.763 (0.630, 0.923) 0.00536	0.773 (0.649, 0.920) 0.00380	0.768 (0.656, 0.899) 0.00103
D5	0.714 (0.572, 0.890) 0.00279	0.677 (0.553, 0.830) 0.00017	0.665 (0.553, 0.799) < 0.0001	0.674 (0.571, 0.796) < 0.0001
D6	0.598 (0.479, 0.748) < 0.0001	0.730 (0.598, 0.891) 0.00198	0.620 (0.514, 0.748) < 0.0001	0.575 (0.485, 0.681) < 0.0001
D7	0.737 (0.590, 0.921) 0.00724	0.693 (0.559, 0.859) 0.00083	0.672 (0.553, 0.817) < 0.0001	0.667 (0.561, 0.792) < 0.0001
D8	0.646 (0.514, 0.813) 0.00019	0.646 (0.519, 0.805) 0.00010	0.613 (0.502, 0.749) < 0.0001	0.616 (0.516, 0.735) < 0.0001
D9	0.625 (0.495, 0.789) < 0.0001	0.584 (0.465, 0.734) < 0.0001	0.587 (0.479, 0.720) < 0.0001	0.584 (0.486, 0.702) < 0.0001
D10	0.567 (0.444, 0.725) < 0.0001	0.563 (0.443, 0.715) < 0.0001	0.524 (0.421, 0.652) < 0.0001	0.523 (0.430, 0.637) < 0.0001

Model I was sensitivity analysis in participants without BMI ≥ 28 kg/m^2^ (*N* = 20,431). We adjusted age, gender, height, SBP, DBP, FPG, BUN, TG, HDL-c, LDL-c, ALT, AST, family history of diabetes, smoking and drinking status

Model II was a sensitivity analysis performed on never smoker participants (*N* = 17,784). We adjusted age, gender, height, SBP, DBP, FPG, BUN, TG, HDL-c, LDL-c, ALT, AST, family history of diabetes, and drinking status

Model III was a sensitivity analysis performed on never drinker participants (*N* = 19,704). We adjusted age, gender, height, SBP, DBP, FPG, BUN, TG, HDL-c, LDL-c, ALT, AST, family history of diabetes, and smoking status

Model IV was sensitivity analysis in participants without adjusting smoking and drinking status (*N* = 24,506). We adjusted age, gender, height, SBP, DBP, FPG, BUN, TG, HDL-c, LDL-c, ALT, AST, family history of diabetes

*HR* Hazard ratios, *CI* Confidence, *Ref* Reference, *Cre/BW ratio* Creatinine to body weight ratio

Since smoking and alcohol status had about 70 percent of missing data, these data might not be suitable as covariates. In other sensitivity analyses, we excluded drinking and smoking status from the multivariate model. It was still similar to the previous results (HR = 0.446, 95% CI:0.362 to 0.549). The results obtained from sensitivity analysis indicated the well-robustness of our findings (Table 5).

### Cox proportional hazards regression model with cubic spline functions to address non-linearity

**Fig. 7:**
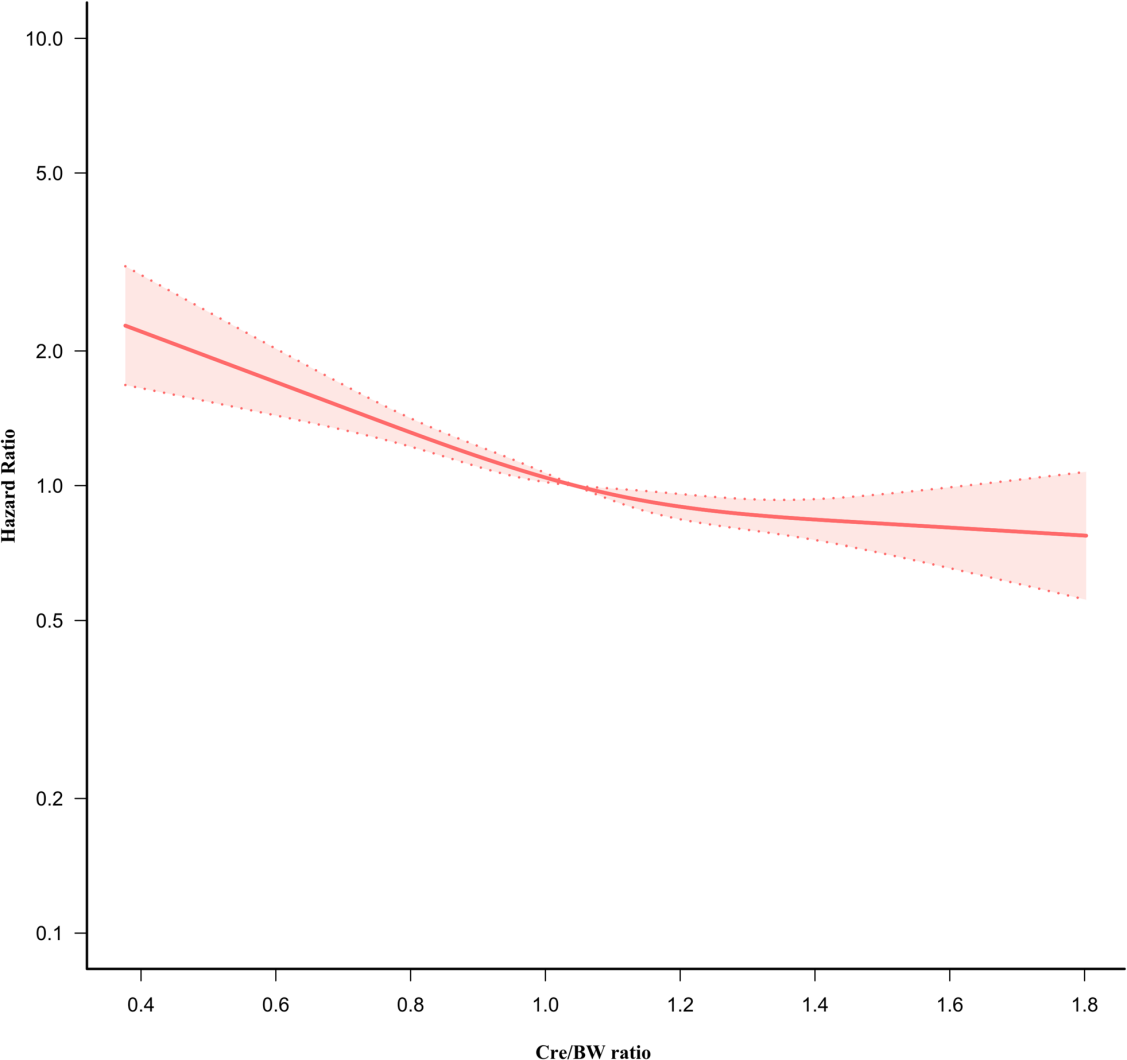
The non-linear relationship between the Cre/BW ratio and the risk of diabetes. Figure 7. We used a Cox proportional hazards regression model with cubic spline functions to evaluate the relationship between the Cre/BW ratio and diabetes risk. The relationship between the Cre/BW ratio and diabetes showed an L-shaped curve with an inflection point of 1.072 umol/L/kg

We discovered that the Cre/BW ratio and incident diabetes relationship in pre-diabetic patients was also non-linear using Cox proportional hazards models with cubic spline functions and smooth curve fitting (Fig. 7). We used a basic Cox proportional-hazards regression model to fit the data based on the sensitivity analysis, and we determined the best-fitting model by calculating log-likelihood ratios. In our analysis, we discovered that the *P* value for the log-likelihood ratio test was less than 0.05. We first determined the inflection point to be 1.072 umol/L/kg using a recursive technique. We then used two-piecewise Cox proportional-hazards regression models to calculate the HR and CI on either side of the inflection point. At the inflection point on the left side, the effect size and 95%CI were 0.294 (0.208, 0.414), respectively. At the inflection point on the right side, they were 0.712 (0.492, 1.029), respectively (Table 6).

**Table 6:** The result of the two-piecewise Cox regression model

Incident diabetes	Model I(HR,95%CI, P)
Fitting model by standard Cox regression	0.445 (0.361, 0.548) < 0.0001
Fitting model by two-piecewise Cox regression	
Inflection point of the Cre/BW ratio	1.072
≤ Inflection point	0.294 (0.208, 0.414) < 0.0001
> Inflection point	0.712 (0.492, 1.029) 0.0706
P for log-likelihood ratio test	0.003

We adjusted age, gender, height, SBP, DBP, FPG, BUN, TG, HDL-c, LDL-c, ALT, AST, family history of diabetes, smoking and drinking status

*HR* Hazard ratios, *CI* Confidence, *Ref* Reference, *Cre/BW ratio* Creatinine to body weight ratio

To explore the non-linear relationship between the Cre/BW ratio and diabetes in pre-diabetic and non-pre-diabetic participants, we further investigated the non-linear relationship between the Cre/BW ratio and diabetes in people without pre-diabetes. It was found that the relationship between the Cre/BW ratio and diabetes remained non-linear in the population without pre-diabetes (Fig. S1). After controlling for covariates, the point of inflection for the Cre/BW ratio was 1.068 umol/L/kg. At the inflection point on the left side, the HR and 95%CI were 0.030 (0.018, 0.049), respectively. At the inflection point on the right side, they were 0.650 (0.382, 1.106), respectively (Table S3).

### The results of subgroup analyses

The grouping of variables such as Gender, BMI, SBP, family history of diabetes, smoking, and alcohol use status did not affect the relationship between Cre/BW ratio and the risk of progression to diabetes in prediabetes (Table 7). In contrast, significant interactions were detected in variables such as age, DBP, and TG.

**Table 7:** Effect size of the Cre/BW ratio on incident diabetes in prespecified and exploratory subgroups

Characteristic	No of participants	HR (95%CI)	*P* value	P for interacion
Age(years)				0.0002
20 to < 30	1431	0.036 (0.005, 0.245)	0.0007	
30 to < 40	5836	0.192 (0.097, 0.380)	< 0.0001	
40 to < 50	5366	0.325 (0.196, 0.539)	< 0.0001	
50 to < 60	5702	0.545 (0.376, 0.791)	0.0014	
60 to < 70	4198	0.689 (0.462, 1.026)	0.0666	
≥ 70	1973	0.786 (0.476, 1.298)	0.3475	
Gender				0.3762
Male	16,232	0.467 (0.367, 0.595)	< 0.0001	
Female	8264	0.382 (0.259, 0.563)	< 0.0001	
BMI(kg/m^2^)				0.5359
< 18.5	437	0.308 (0.014, 6.724)	0.4542	
≥ 18.5, < 24	9544	0.517 (0.345, 0.776)	0.0014	
≥ 24, < 28	10,450	0.749 (0.533, 1.051)	0.0944	
≥ 28	4075	0.593 (0.367, 0.958)	0.0328	
Smoking status				0.2491
Never smoker	17,784	0.462 (0.361, 0.591)	< 0.0001	
Ever smoker	1110	0.215 (0.088, 0.526)	0.0008	
Current smoker	5612	0.461 (0.314, 0.676)	< 0.0001	
Drinking status				0.8016
Never drinker	19,704	0.453 (0.360, 0.570)	< 0.0001	
Ever drinker	3846	0.380 (0.222, 0.651)	0.0004	
Current drinker	956	0.508 (0.204, 1.266)	0.1459	
Family history of diabetes				0.7916
No	23,887	0.448 (0.362, 0.554)	< 0.0001	
Yes	619	0.393 (0.150, 1.031)	0.0576	
SBP(mmHg)				0.4140
< 140	19,302	0.429 (0.335, 0.550)	< 0.0001	
≥ 140	2504	0.506 (0.362, 0.705)	< 0.0001	
DBP(mmHg)				0.0358
< 90	20,966	0.404 (0.322, 0.508)	< 0.0001	
≥ 90	3540	0.675 (0.437, 1.041)	0.0756	
TG(mmol/L)				0.0286
< 1.7	14,958	0.373 (0.281, 0.495)	< 0.0001	
≥ 1.7	9548	0.570 (0.430, 0.755)	< 0.0001	

Note 1: Above model adjusted for age, gender, height, SBP, DBP, FPG, BUN, TG, HDL-c, LDL-c, ALT, AST, family history of diabetes, smoking and drinking status

Note 2: In each case, the model is not adjusted for the stratification variable

Specifically, a stronger association between the Cre/BW ratio and diabetes was observed in the participants who were younger than 30 years (HR = 0.036,95%CI:0.005–0.245) and 30–50 years (HR = 0.192,95%CI:0.097–0.380; HR = 0.325,95%CI:0.196–0.539), as well as among those with TG < 1.7 mmol/L(HR = 0.373,95%CI:0.281–0.495) and DBP < 90 mmHg (HR = 0.373, 95%CI: 0.281–0.495). In contrast, the association of the Cre/BW ratio with the risk of incident diabetes was attenuated among participants who were above 50 years of age (HR = 0.545, 95%CI: 0.376–0.791; HR = 0.689,95%CI:0.462–1.206; HR = 0.786,95%CI:0.476–1.298), and among those with DBP ≥ 90 mmHg (HR = 0.675,95%CI:0.437–1.041) and TG ≥ 1.7 mmol/L (HR = 0.570, 95%CI:0.430–0.755).

## Discussion

The purpose of our retrospective cohort study was to investigate the relationship between the Cre/BW ratio and diabetes in pre-diabetic patients. We discovered that increasing the Cre/BW ratio was associated with a considerably lower risk of diabetes. Furthermore, a threshold effect curve was discovered, and different associations between the Cre/BW ratio and diabetes risk were discovered on both sides of the inflection point. In addition, as potential moderators of the relationship between the Cre/BW ratio and pre-diabetes, age, TG, and DBP were found to be significant, as significantly stronger associations were observed in participants with age < 50 years, TG < 1.7 mmol/L, and DBP < 90 mmHg, while significantly weaker associations were detected in age > 50 years, TG ≥ 1.7 mmol/L, DBP ≥ 90 mmHg.

In a Japanese study, the actual 5-year transition rate from pre-diabetes to diabetes was 8.5% [40]. In American pre-diabetic patients aged 70–79, the 7-year incidence of developing diabetes was 10.6% [41]. In the current investigation, over a median follow-up period of 2.89 years, the incidence of diabetes among patients with pre-diabetes was 10.25%. Variations in the age and ethnicity of the participants may bring about these variations in the incidence of diabetes among these pre-diabetic patients. It is important to note that pre-diabetic people have a high chance of acquiring diabetes, according to all research. Therefore, it is crucial to actively look for additional risk factors for developing diabetes from pre-diabetes.

The Cre/BW ratio was found to be inversely associated with the risk of diabetes in a recent Japanese study (HR = 0.84, 95% CI 0.80–0.88 for men and 0.88, 0.81–0.96 for women) in the general population [16] after adjusting age, FPG, drinking status, exercise, and smoking status. A Chinese study also found an inverse association between the Cre/BW ratio and the risk of diabetes in the general population (HR = 0.268, 95%CI 0.229–0.314) after adjusting confounding variables. They also found a negative non-linear connection between the Cre/BW ratio and incident diabetes [20]. By Cox proportional hazards regression model analysis, our study found that the decline in the Cre/BW ratio was strongly associated with an increased risk of diabetes, which was consistent with previous findings. At the same time, it is essential to point out that, unlike the above two studies, our study focused on pre-diabetic adults at higher risk of progression to diabetes. For this population, it is of great significance to delay its progression to diabetes and even propose to switch to normoglycemia with aggressive intervention. This is the first study to examine the relationship between Cre/BW ratio and diabetes risk in patients with pre-diabetes. Identifying the Cre/BW ratio as a protective factor for developing diabetes in pre-diabetes and clarifying the association between them will benefit diabetes prevention in patients with pre-diabetes. Also, the HRs of the relationship between creatinine body weight ratio and diabetes were different in our study from that of Chen et al. [20].

Concurrently, our sensitivity analysis found that the relationship remained stable among participants who never smoked or drank and those who did not have a BMI > 28 kg/m^2^. We also found that the negative association between the Cre/BW ratio and diabetes remained stable when the multiple regression equation did not adjust smoking and drinking status. The efforts indicated above supported the stability of the association between the Cre/BW ratio and diabetes risk.

The following are some potential explanations for the connection between the Cre/BW ratio and the progression from pre-diabetes to diabetes. Although there are no published studies on Cre/BW ratio and progression from pre-diabetes to diabetes, it is clear that insulin resistance is strongly associated with progression from pre-diabetes to diabetes [8, 42, 43]. It is generally known that muscle mass absorbs 80%-90% of blood glucose when the body is in a hyperinsulinemia-euglycemia state [44]. The ability to absorb glucose from the blood can be decreased with less muscle mass [45]. Moreover, insulin resistance, a major pathogenic mechanism of diabetes, may mediate the association between low muscle mass and incident diabetes [46]. The proportion of muscle mass to body weight is crucial since both an increase in muscle mass and fat mass could result in weight gain [47]. According to numerous research, weight-adjusted appendicular skeletal muscle mass has been linked to IR and cardiometabolic risk factors [12, 48–51]. Moreover, weight-adjusted appendicular skeletal muscle mass is associated with incident diabetes [14], metabolic syndrome [52], and NAFLD [53, 54]. This is due to the fact that a low skeletal muscle mass index (SMI) is linked to an increase in visceral fat, which in turn is connected to diabetes [55]. Cre is an alternative skeletal muscle mass marker; its levels positively correlate with skeletal muscle mass [56, 57]. Consequently, the weight-adjusted SMI was positively correlated with the Cre/BW ratio. Therefore, we propose the possibility that the relationship between Cre/BW ratio and progression from pre-diabetes to diabetes is mediated through insulin resistance.

Furthermore, the present investigation discovered for the first time a non-linear link between the Cre/BW ratio and the risk of diabetes in pre-diabetic patients. To explain the non-linear association, this study used a two-piecewise Cox proportional hazards regression model. The findings revealed a non-linear relationship and saturation effect between the Cre/BW ratio and pre-diabetic patients' probability of developing diabetes. After controlling for covariates, the point of inflection for the Cre/BW ratio was 1.072 umol/L/kg. A 1 umol/L/kg rise in the Cre/BW ratio was related to a 70.6% reduction in the risk of diabetes when the ratio was below 1.072 umol/L/kg. As we mentioned above, when the Cre/BW ratio was < 1.072 umol/L/kg, as the Cre/BW ratio increases, the skeletal muscle mass increases accordingly, so that the risk of developing insulin resistance or diabetes decreases accordingly. However, at the same time, we also found that the risk of diabetes did not reduce significantly with the increase of the Cre/BW ratio, when the Cre/BW ratio was above 1.072 umol/L/kg. The possible reason is that although Cre is an alternative marker of skeletal muscle mass, at the same time, it is also an indicator to reflect the state of renal function. Creatinine levels were influenced by renal functional status. Our previous study found that a decline in the glomerular filtration rate (GFR) was strongly associated with an increased risk of diabetes [58]. Chronic kidney disease (CKD) was a crucial and independent predictor of diabetes, according to another population-based cohort study in Taiwan (adjusted HR 1.204; 95% CI 1.11, 1.31) [59]. A recent study also showed that a decrease in GFR was associated with an increase in IR in 4680 American participants without diabetes [60]. This might be the reason when the Cre/BW ratio was ≥ 1.072 umol/L/kg, the risk of diabetes no longer decreased accordingly with the Cre/BW ratio increased. These results indicate that the Cre/BW ratio may be a viable predictor for individuals who wish to prevent diabetes by losing weight. Therefore, patients with pre-diabetes can try to control the Cre/BW ratio above 1.072 umol/L/kg through various treatment modalities, then the risk of progression to diabetes may be significantly reduced. Excellent clinical relevance can be derived from the identification of a non-linear association between the Cre/BW ratio and diabetes in pre-diabetic patients. In patients with pre-diabetes, it promotes clinical consultation and offers a reference for decision-making that is optimized for diabetes prevention. In addition, although the inflection points for the Cre/BW ratio were relatively similar in those with and without pre-diabetes (1.072 and 1.068 umol/L/kg), there were significant differences in the effect values for the relationship between Cre/BW ratio and diabetes on both sides of the inflection points. A 1 umol/L/kg rise in the Cre/BW ratio was related to a 70.6% reduction in the risk of diabetes in participants with pre-diabetes when the ratio was below 1.072 umol/L/kg. However, a 1 umol/L/kg rise in the Cre/BW ratio was related to a 97% reduction in the risk of diabetes in participants without pre-diabetes when the ratio was below 1.068 umol/L/kg. This result further suggests that patients with pre-diabetes are at high risk of progression to diabetes and that reducing the risk of developing diabetes by intervening in the Cre/BW ratio is not as significant as in non-prediabetic patients. Therefore, patients with pre-diabetes must be more aggressively sought and controlled for associated risk factors to reduce the risk of progression from pre-diabetes to diabetes.

We have outlined some of our study's advantages below. (1) Our study's overall sample size was large, which is a strength. (2) To the best of our knowledge, this is the first study to investigate the relationship between the Cre/BW ratio and the risk of diabetes in Chinese pre-diabetic individuals. (3) In this study, non-linearity is investigated and further explained. This study significantly improves upon earlier research. (4) In this study, missing data were handled using multiple imputations. Using multiple imputations could increase statistical power and reduce any potential bias brought on by the absence of covariate information. (5) Since this is an observational study, it is vulnerable to potential confounding. We minimized residual confounding by using strict statistical adjustment. (6) A series of sensitivity analyses were carried out throughout this study to ensure the accuracy of the findings (conversion of target-independent variable form, subgroup analysis, using cubic splines to insert the continuity covariate into the equation as a curve, calculating E-values to investigate the possibility of unmeasured confounding, and reanalyzing the association between the Cre/BW ratio and diabetes among participants who never smoked or drank, and among those who did not have a BMI > 28 kg/m^2^).

Our research has the following flaws that must be addressed: First, because the design of this study is observational, we cannot determine the exact causal relationship. Second, as with all observational studies, even while known potential confounders such as TG, age, FPG, and BUN were controlled, uncontrolled or unmeasured confounders may still have been present. In spite of this, the authors estimated the E-value to quantify the influence of unmeasured confounders and concluded that they were unlikely to account for the results. Third, diabetes was defined as an FPG ≥ 7.00 mmol/L and/or self-reported diabetes throughout the follow-up period rather than a 2-h oral glucose tolerance test or glycosylated hemoglobin level, which may underestimate the incidence of diabetes. Besides, there is a general issue with the outcome variable, in that it is not purely objective and people with certain characteristics may be followed up more closely and hence diagnosed earlier. In addition, diabetes's type remained unknown. Since T2DM accounts for more than 90% of all diabetes cases in China, it is the most prevalent kind of diabetes [61]. Our findings are, therefore, representative of T2DM patients. Fourth, the information from the original study did not include cases of diabetes during the first 2 years of follow-up. For such a large sample of participants, possible deaths during follow-up and diabetes observed in the first 2 years were inevitable. In the future, we can consider designing our studies and collecting the observed cases of diabetes throughout the follow-up period. Fifth, what readers need to know is whether they can use the Cre/BW ratio to predict subsequent diabetes. Therefore, a score based the ratio and other covariates is necessary. In the future we can collect our database and build a prediction model for progression of pre-diabetes to diabetes by Cre/BW ratio and other indicators. Sixth, we only measured the Cre/BW ratio at baseline and did not account for changes in the Cre/BW ratio over time. As a result, future studies should include as many variables as possible, including information on changes in the Cre/BW ratio during the study period. Sixth, the decile analysis may be confusing, since it is only when adding the blood covariates does the relationship of risk to Cre/BW ratio become non-monotonic. As the confounding factors change, the relationship between the Cre/BW ratio and the risk of progression to diabetes from pre-diabetes may change accordingly. In future studies, we will collaborate with other researchers to design cohort studies and collect more variables to observe changes in the relationship between the Cre/BW ratio and the risk of progression to diabetes from pre-diabetes by adjusting for various confounders. Finally, in this study, pre-diabetes was only recognized by the researchers in the subjects with impaired fasting glucose levels, which could have resulted in a missed diagnosis [62].

## Conclusion

This study indicates a negative and non-linear association between the Cre/BW ratio and incident diabetes in pre-diabetic Chinese individuals. A saturating effect exists between the Cre/BW ratio and diabetes risk in pre-diabetic patients. When the Cre/BW ratio is < 1.072 umol/L/kg, there was a significant negative association with the risk of progression from pre-diabetes to diabetes. This result will offer clinicians a reference for aggressive weight loss and muscle mass increase in pre-diabetic patients. From a treatment perspective, it makes sense to increase the Cre/BW ratio above the inflection point by aggressive intervention to aggressive muscle mass gain and weight loss.

### Supplementary Information

**Additional file 1:** **Table S1.** Collinearity diagnostics steps. **Table S2.** Relationship between Cre, weight and the incident diabetes in different models. **Table S3.** The result of the two-piecewise Cox regression model in participants without prediabetes. **Figure S1.** The non-linear relationship between the Cre/BW ratio and the risk of diabetes in participants without prediabetes.

## Data Availability

The data used in the study is from a publicly available database. Data could be downloaded from the 'DATADRYAD' database (https://datadryad.org/stash).

## Abbreviations

Cre/BW: Creatinine to body weight
DM: Diabetes mellitus
Cre: Creatinine
IDF: International Diabetes Federation
NAFLD: Non-alcoholic fatty liver disease
HTG: Hypertriglyceridemia
IGT: Impaired glucose tolerance
SMI: Skeletal muscle mass index
SBP: Systolic blood pressure
Scr: Serum creatinine
T2DM: Type 2 diabetes mellitus
ALT: Alanine aminotransferase
BMI: Body mass index
TG: Triglyceride
DBP: Diastolic blood pressure
VIF: Variance inflation factor
GFR: Glomerular filtration rate
LDL-c: Low-density lipid cholesterol
HDL-c: High-density lipoprotein cholesterol
CKD: Chronic kidney disease
AST: Aspartate aminotransferase
FPG: Fasting plasma glucose
TC: Total cholesterol
BUN: Blood urea nitrogen
IFG: Impaired fasting glucose
GAM: Generalized additive models
HR: Hazard ratios
Ref: Reference
CI: Confidence intervals
MAR: Missing-at-random
IR: Insulin resistance
SD: Standard deviation
