# Correction: The association between creatinine to body weight ratio and the risk of progression to diabetes from pre-diabetes: a 5-year cohort study in Chinese adults

**DOI:** 10.1186/s12902-023-01531-y

**Published:** 2024-01-02

**Authors:** Tong Li, Changchun Cao, Xuan Xuan, Wenjing Liu, Xiaohua Xiao, Cuimei Wei

**Affiliations:** 1 Department of Nephrology, Shenzhen Second People’s Hospital, 518000 Shenzhen, Guangdong Province China; 2 Department of Nephrology, The First Affiliated Hospital of Shenzhen University, 518000 Shenzhen, Guangdong Province China; 3 Department of Rehabilitation, Shenzhen Dapeng New District Nan’ao People’s Hospital, 518000 Shenzhen, Guangdong Province China; 4 Department of Rheumatology, Shenzhen Second People’s Hospital, 518000 Shenzhen, Guangdong Province China; 5 Department of Rheumatology, The First Affiliated Hospital of Shenzhen University, 518000 Shenzhen, Guangdong Province China; 6 Department of Geriatrics, Shenzhen Second People’s Hospital, No.3002 Sungang Road, Futian District, 518000 Shenzhen, Guangdong Province China; 7 Department of Geriatrics, The First Affiliated Hospital of Shenzhen University, 518000 Shenzhen, Guangdong Province China

**Correction:** ***BMC Endocr Disord*** **23, 266 (2023)**

10.1186/s12902-023-01518-9

Following publication of the original article [1], the authors reported that the funding information was mistakenly missing in the article.

The correct Funding section should read:

This study were supported by Shenzhen Key Medical Discipline Construction Fund (SZXK009), Shenzhen Municipal Science and Technology Innovation Commission for Sustainable Development (KCXFZ20201221173213036), Shenzhen High-level Hospital Construction Fund.

The original article [1] has been updated.
