# Supplementary material for: The association between creatinine to body weight ratio and the risk of progression to diabetes from pre-diabetes: a 5-year cohort study in Chinese adults
Source: BMC Endocr Disord. 2023 Dec 4;23:266. doi: 10.1186/s12902-023-01518-9 (PMC10694873; doi:10.1186/s12902-023-01518-9)
Supplement: Supplementary file 1 — Additional file 1: Table S1. Collinearity diagnostics steps. Table S2. Relationship between Cre, weight and the incident diabetes in different models. Table S3. The result of the two-piecewise Cox regression model in participants without prediabetes. Figure S1. The non-linear relationship between the Cre/BW ratio and the risk of diabetes in participants without prediabetes. [file 12902_2023_1518_MOESM1_ESM.docx]

**The association between creatinine to body weight ratio and** **the risk of progression to diabetes from pre-diabetes: a 5-year cohort study in Chinese adults**

**Running title:** **Cre/BW ratio and diabetes**

**Tong Li^1,2#^,** **Changchun Cao^3#^,** **Xuan Xuan^4,5^, Wenjing Liu^6,7^, Xiaohua Xiao^6,7*^, Cuimei Wei^6,^****^7*^,**

^1^Department of Nephrology, Shenzhen Second People’s Hospital, Shenzhen 518000, Guangdong Province, China

^2^Department of Nephrology, The First Affiliated Hospital of Shenzhen University, Shenzhen 518000, Guangdong Province, China

^3^Department of Rehabilitation, Shenzhen Dapeng New District Nan’ao People’s Hospital, Shenzhen 518000, Guangdong Province, China

^4^Department of Rheumatology, Shenzhen Second People’s Hospital, Shenzhen 518000, Guangdong Province, China

^5^Department of Rheumatology, The First Affiliated Hospital of Shenzhen University, Shenzhen 518000, Guangdong Province, China

^6^Department of Geriatrics, Shenzhen Second People’s Hospital, Shenzhen 518000, Guangdong Province, China

^7^Department of Geriatrics, The First Affiliated Hospital of Shenzhen University, Shenzhen 518000, Guangdong Province, China

**^#^Tong Li and Changchun Cao have contributed equally to this work.**

***Corresponding author**

Xiaohua Xiao

Department of Geriatrics,

Shenzhen Second People’s Hospital,

No.3002 Sungang Road, Futian District,

Shenzhen 518000,

Guangdong Province,

China

Tel: +86-755-83366388

E-mail: tu_xi8888@163.com

***Corresponding authors**

**Cuimei Wei**

Department of Geriatrics,

Shenzhen Second People’s Hospital,

No.3002 Sungang Road, Futian District,

Shenzhen 518000,

Guangdong Province,

China

Tel: +86-755-83366388

E-mail: weicuimei2022@126.com

**Table S1. Collinearity diagnostics steps.**

| Variable | VIF  Step 1 | Step 2 |
| --- | --- | --- |
|  |  |  |
| Gender | 2.3 | 2.3 |
| Age(years) | 1.4 | 1.4 |
| Smoking status | 1.3 | 1.3 |
| Drinking status | 1.2 | 1.2 |
| ALT(U/L) | 3.5 | 3.5 |
| AST(U/L) | 3.3 | 3.3 |
| Family history of diabetes | 1.0 | 1.0 |
| BUN(mmol/L) | 1.1 | 1.1 |
| FPG(mmol/L) | 1.1 | 1.1 |
| TC(mmol/L) | 6.4 | NA |
| TG(mmol/L) | 1.8 | 1.2 |
| HDL-c(mmol/L) | 1.3 | 1.1 |
| LDL-c(mmol/L) | 5.3 | 1.1 |
| Height(cm) | 2.0 | 2.0 |
| SBP(mmHg) | 1.9 | 1.9 |
| DBP(mmHg) | 1.8 | 1.8 |

SBP, Systolic blood pressure; DBP, Diastolic blood pressure; ALT, Alanine aminotransferase; AST, Aspartate aminotransferase; TC, Total cholesterol; TG, Triglyceride; HDL-c, High-density lipoprotein cholesterol; LDL-c, Low-density lipid cholesterol; BUN, Serum urea nitrogen; FPG, Fasting plasma glucose;

Abbreviation: VIF: variance inflation factor; VIF = 1/(1-R^2^).

Note: The variables with VIF>5 will be regarded as collinear variables and cannot be included in the multiple regression model.

**Table S2. Relationship between Cre, weight and the incident diabetes in different models**

| Exposure | Model I(HR,95%CI,P) | Model II(HR,95%CI,P) | Model III(HR,95%CI,P) |
| --- | --- | --- | --- |
| Log(Cre) | 1.164 (0.756, 1.791) 0.49095 | 0.154 (0.086, 0.276) <0.00001 | 0.542 (0.298, 0.985) 0.04452 |
| Log(weight) | 44.110 (25.934, 75.023) <0.00001 | 364.686 (176.815, 752.178) <0.00001 | 60.780 (28.568, 129.314) <0.00001 |

Model I: we did not adjust other covariates.

Model II: we adjust age, gender, height, SBP, DBP, family history of diabetes, smoking and drinking status.

Model III: we adjust age, gender, height, SBP, DBP, FPG, BUN, TG, HDL-c, LDL-c, ALT, AST, family history of diabetes, smoking and drinking status..

HR, Hazard ratios; CI: confidence, Ref: reference;

**Table S3. The result of the two-piecewise Cox regression model in participants without prediabetes**

| Incident diabetes | Model I(HR,95%CI, P ) |
| --- | --- |
| Fitting model by standard Cox regression | 0.122 (0.089, 0.167) <0.0001 |
| Fitting model by two-piecewise Cox regression |  |
| Inflection point of the Cre/BW ratio | 1.068 |
| ≤Inflection point | 0.030 (0.018, 0.049) <0.0001 |
| >Inflection point | 0.650 (0.382, 1.106) 0.1125 |
| P for log-likelihood ratio test | <0.001 |

We adjusted age, gender, height, SBP, DBP, FPG, BUN, TG, HDL-c, LDL-c, ALT, AST, family history of diabetes, smoking and drinking status.

HR, Hazard ratios; CI: confidence, Ref: reference; Cre/BW ratio, creatinine to body weight ratio

**Figure S1. The non-linear relationship between the Cre/BW ratio and the risk of diabetes in participants without prediabetes.**


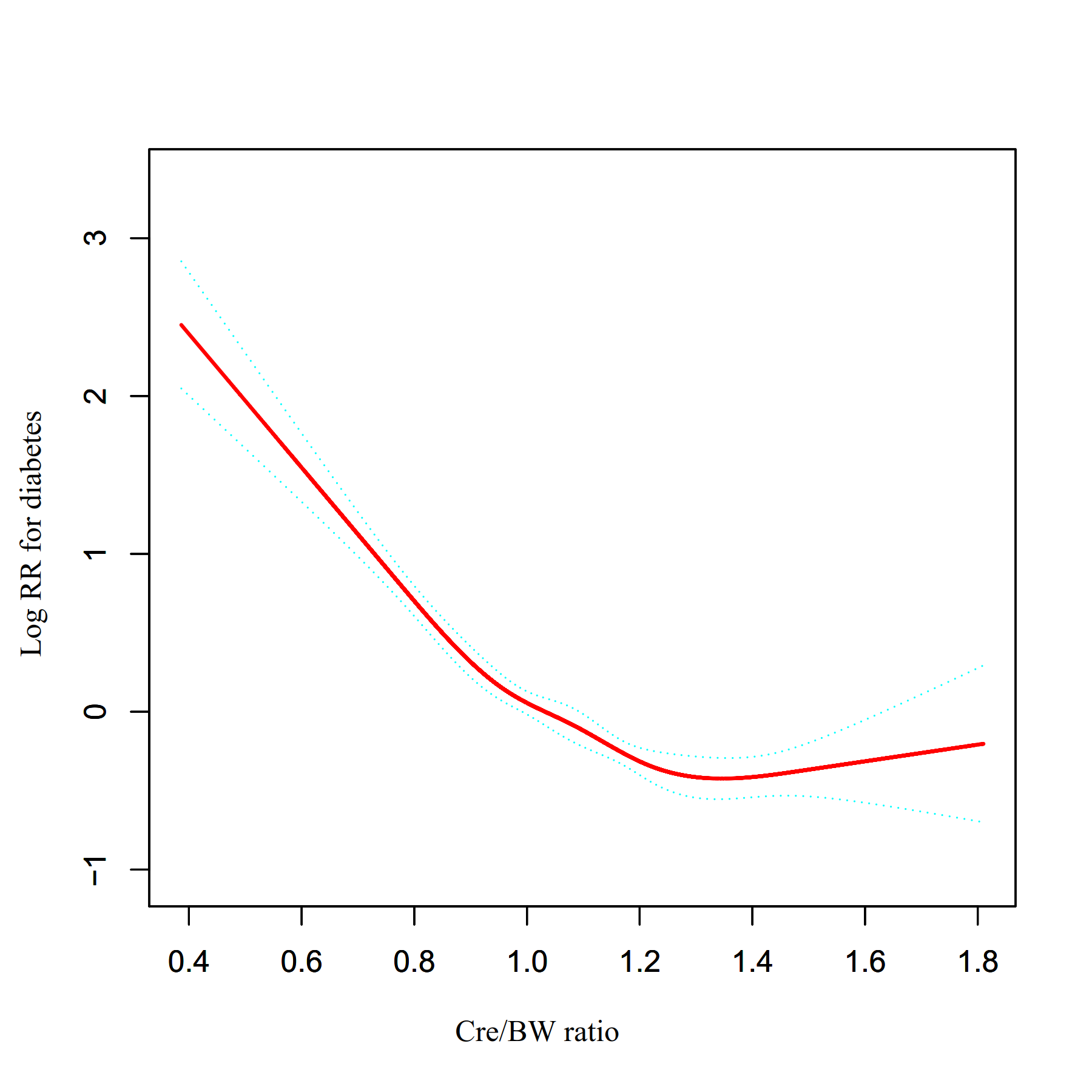


Figure S1. We used a Cox proportional hazards regression model with cubic spline functions to evaluate the relationship between the Cre/BW ratio and diabetes risk in participants without prediabetes. The relationship between the Cre/BW ratio and diabetes also showed a L-shaped curve with an inflection point of 1.068 umol/L/kg.
